# Hematological Questions in Personalized Management of COVID-19 Vaccination

**DOI:** 10.3390/jpm13020259

**Published:** 2023-01-30

**Authors:** Tingting Wu, Junying Li, Yu Hu, Liang V. Tang

**Affiliations:** Institute of Hematology, Union Hospital, Tongji Medical College, Huazhong University of Science and Technology, Wuhan 430022, China

**Keywords:** COVID-19, SARS-CoV-2, vaccine, hematological events, hematological malignancies

## Abstract

Coronavirus disease 2019 (COVID-19), caused by severe acute respiratory syndrome coronavirus 2 (SARS-CoV-2), has been causing a worldwide pandemic since 2019. Many vaccines have been manufactured and have shown promising results in reducing disease morbidity and mortality. However, a variety of vaccine-related adverse effects, including hematological events, have been reported, such as thromboembolic events, thrombocytopenia, and bleeding. Moreover, a new syndrome, vaccine-induced immune thrombotic thrombocytopenia, following vaccination against COVID-19 has been recognized. These hematologic side effects have also raised concerns about SARS-CoV-2 vaccination in patients with preexisting hematologic conditions. Patients with hematological tumors are at a higher risk of severe SARS-CoV-2 infection, and the efficacy and safety of vaccination in this group remain uncertain and have raised attention. In this review, we discuss the hematological events following COVID-19 vaccination and vaccination in patients with hematological disorders.

## 1. Introduction

Coronavirus disease 2019 (COVID-19) is caused by a highly infectious virus named severe acute respiratory syndrome coronavirus 2 (SARS-CoV-2). SARS-CoV-2 is a single-stranded RNA virus. The virus has rapidly spread and caused an unprecedented worldwide pandemic. SARS-CoV-2 can affect not only the respiratory system but also various organs, such as the cardiovascular system, neurological system, and gastrointestinal tract. In addition, atypical clinical manifestations of COVID-19 include cutaneous manifestations and olfactory/gustatory disorders [1]. Infected individuals can present with clinical courses ranging from mild to severe and even death in some cases. According to the data released by the World Health Organization (WHO), as of 12 December 2022, there have been 645,084,824 confirmed cases of COVID-19 and 6,633,118 deaths [2]. To date, several effective treatments against COVID-19 have been explored, including dexamethasone, monoclonal antibodies (tocilizumab), and other small molecules targeting specific signals and functions. Small molecules such as remdesivir, paxlovid, and baricitinib have already been approved in many countries [3,4]. Vaccination is an effective strategy to control the pandemic. Many vaccines have been manufactured and have shown promising results in reducing disease morbidity and mortality. By 10 December 2022, a total of 13,008,033,382 vaccine doses have been administered. Approximately 5,449,470,580 individuals have received at least one dose of a vaccine, and 5,002,932,205 persons have received full vaccination [2]. Although the safety and efficacy of available vaccines have been proven in clinical trials, severe adverse effects after COVID-19 vaccination have been reported following administration on a large scale, including hematologic vaccine-related adverse events. Furthermore, these hematologic side effects have also raised concerns about SARS-CoV-2 vaccination in patients with preexisting hematologic conditions.

In this review we discuss the adverse events following COVID-19 vaccination, focusing on associated hematological questions. These adverse effects include thrombotic events, thrombocytopenia (vaccine-induced immune thrombotic thrombocytopenia, immune thrombocytopenia, and thrombotic thrombocytopenic purpura), hemorrhagic events, and other rare hematological events related to COVID-19 vaccination. Moreover, we discuss COVID-19 vaccination in individuals with preexisting hematological disorders, in particular the disorders which have been associated with adverse reactions after vaccination. 

## 2. Brief Review of COVID-19 Vaccines

Many types of vaccines against SARS-CoV-2 have been produced, and commonly used COVID-19 vaccines at present are described as follows. According to the vaccine platform, COVID-19 vaccines can be classified into inactivated, adenovirus-based, mRNA, and protein subunit vaccines. Inactivated vaccines reserve the entire virus as an immunogen after inactivating the virus via chemical reagents, which triggers immune responses and leads to the production of a wide spectrum of antibodies. Inactivated vaccines include BBIBP-CorV (Sinopharm), WIBP-CorV (Sinopharm), CoronaVac (Sinovac Biotech), etc. Viral vector vaccines are based on engineered viral delivery systems carrying nucleic acid that encodes viral proteins or polypeptides [5]. The ChAdOx1 nCoV-19 vaccine uses recombinant chimpanzee adenovirus 25 vector containing genetic information for the full-length spike protein of SARS-CoV-2. Other viral vectors used include the combination of human Ad5 and Ad26 vectors. Viral vector-based vaccines trigger Th1 cell responses. mRNA vaccines carry genetic material encoding the viral antigen. The two most commonly used mRNA vaccines are BNT162b2 and mRNA-1273, both encapsulated by lipid nanoparticles. The lipid nanoparticles’ capsulation of mRNA vaccines facilitates mRNA transportation, thus, inducing strong immune responses. In addition, protein subunit vaccines are based on cell-expressing systems to produce viral proteins. The recombinant viral proteins and vaccine adjuvant can induce immune responses [6]. Commonly used COVID-19 vaccines are summarized in Table 1. 

Currently available vaccines that have passed phase III trials have shown great efficacy in protecting against infection and severe COVID-19 in trials [6]. In real-world settings, the COVID-19 vaccines also present with high effectiveness against SARS-CoV-2 infection (89.1%; 95% CI, 85.6–92.6%), COVID-19 associated hospitalization (97.2%; 95% CI, 96.1–98.3%), and death (99.0%; 95% CI, 98.5–99.6%) [7]. Similar to other vaccines, general adverse effects following COVID-19 vaccination include local injection site reactions (pain, pruritus, induration, erythema, and edema), fever, headache, fatigue, and muscle pain. These symptoms are generally transient and self-limiting. Nevertheless, severe adverse events such as thrombosis, thrombocytopenia, Guillain–Barré syndrome, anaphylaxis, myocarditis, and pericarditis have been recorded. Regarding hematological questions, thrombotic events and thrombocytopenia have been associated with COVID-19 vaccination. In particular, a new syndrome, vaccine-induced immune thrombotic thrombocytopenia (VITT), has been recognized. 

## 3. Hematological Questions following COVID-19 Vaccination

### 3.1. Thrombotic Events

Thromboembolic complications have been reported in patients with COVID-19. The most frequently observed thrombotic events in COVID-19 patients are pulmonary embolism (PE) and deep vein thrombosis (DVT) [8]. With the large-scale vaccination campaign, thrombotic events, such as DVT, PE, cerebral venous sinus thrombosis (CVST), and arterial thrombosis, have been reported following COVID-19 vaccination. Thromboembolism may be associated with or without thrombocytopenia.

In an analysis using the VigiBase, a World Health Organization (WHO) Global Database for Individual Case Safety Reports, the reporting rate of thrombotic events following the BNT162b2, mRNA-1273, and AZD1222 vaccines was 0.21 (95% CI, 0.19–0.22) cases per 1 million vaccinated person-days [9]. A retrospective, nationwide cohort study in Danish on 355,209 individuals vaccinated with AZD1222 or BNT162b2 showed a significant risk difference for DVT (8.35 per 100,000 vaccinations, 95% CI, 0.21 to 16.49) in the recipients of AZD1222 compared with in those with no vaccination; of note, no statistically significant association was seen for thrombotic events and BNT162b2 vaccination [10]. In addition, after analyzing the available data, Matthew Nicholson et al. concluded that mRNA vaccines, including BNT162b2 and mRNA-1273, were not apparently associated with venous thromboembolism (VTE) [11].

VTE following adenovirus-based vaccination had certain characteristics compared to those seen within the control group, including a higher incidence of CVST, more multisite thromboembolism more frequently associated with thrombocytopenia, higher 14-day mortality, and higher incidence of major bleeding events [12]. According to a meta-analysis including 460 thrombotic episodes following COVID-19 vaccination, cerebral venous thrombosis (CVT)/CVST made up the majority of thrombotic events, accounting for 34.6% [13]. In pooled national data from England, Scotland, and Wales, a slightly increased risk of CVST was observed in the 28-day period after the first dose of the ChAdOx1 vaccine, with an incidence rate ratio of 1.93 (95% confidence interval, 1.20–3.11) [14]. Thromboembolic events after COVID-19 vaccination may occur in patients with genetic predispositions for venous thromboembolism, further complicating the complication [15].

Other sites of venous thrombosis include the splenic vein, superior mesenteric vein, portal vein thrombosis [16], central retinal vein [17], superior ocular vein [18], etc.

Regarding arterial thrombosis, it appears to be less common than venous embolism. The clinical features of arterial thrombosis depend on the embolized arteries. Cases of acute ischemic stroke (AIS) have been reported. In one study, 43 AIS patients after COVID-19 vaccination were included, and 51.1% were associated with vaccine-induced immune thrombotic thrombocytopenia, among which 77.2% were female and 68% were aged below 60 [19]. Other vessels involved are the carotid artery, splenic artery, coronary artery, abdominal aorta, peripheral artery, retinal artery [13], etc.

### 3.2. Thrombocytopenia

#### 3.2.1. Vaccine-Induced Immune Thrombotic Thrombocytopenia

Initially described following vaccination with the adenoviral-based vaccines, vaccine-induced immune thrombotic thrombocytopenia is characterized by co-occurring thrombosis and thrombocytopenia. VITT is recognized as a unique clinical syndrome due to a predisposition to cerebral or splanchnic vein thrombosis. In addition, the patients present with positive anti-PF4 antibodies and consumptive coagulopathy disease [20]. It is likely that VITT is exclusively associated with adenovirus-based vaccines (ChAdOx1 and Ad26.COV2.S) [21]. The incidence of VITT is unclear, at approximately one case per 394,000 vaccinations and one case per 282,000 vaccinations after ChadOx1 nCov19 and Ad26.Cov2.S, respectively [22]. In an international network cohort study, an increased risk of venous thrombosis with thrombocytopenia post Ad26.COV2.S vaccine was observed compared with after the BNT162b2 vaccine (pooled calibrated incidence rate ratio, 2.26, 95% confidence interval, 0.93 to 5.52) [23]. 

In a meta-analysis, Ah Young Kim et al. studied the thrombosis patterns and showed a predominance of CVT accounting for 54% of cases, followed by DVT or PE (36%) and splanchnic vein thrombosis (19%) [24]. The mean age of VITT patients was 45.6 years. In addition, VITT was more frequently reported in females, with a rate of 70%. The overall mortality was 32%. Maryam Sharifian-Dorche et al. conducted a study to analyze the clinical features of patients with CVST and VITT after receiving viral vector vaccines. They found that headache was the most common symptom, and the symptom onset occurred between 4 and 19 days following the first dose of the vaccine [25]. Regarding prognostic factors for VITT, in a post hoc analysis including 69 patients [26], the authors revealed that platelet nadir (*p* < 0.001) and chronic medical conditions (χ2 = 25.507, *p* = 0.041) were statistically associated with death. 

#### 3.2.2. Possible Mechanisms of VITT

The mechanism of VITT is not fully understood, and several possible pathological mechanisms have been proposed. Anti-PF4 antibodies are detected in patients with VITT which are typically present in patients with autoimmune heparin-induced thrombocytopenia (aHIT), despite the absence of heparin therapy, suggesting that the pathogenesis of VITT may be similar to that of aHIT [27]. HIT is an immune disorder characterized by a progressive prothrombotic condition after heparin exposure, and anti-PF4 antibodies are diagnostic antibodies [28]. 

Platelet factor 4 is a tetramer with strong positive charges and has a high affinity for heparin. Binding to heparin or other polyanions may induce conformational changes in PF4 and antigenic PF4–heparin formation, thus, leading to the formation of pathogenic antibodies. It is hypothesized that the component of vaccination including polyanionic AdV hexon proteins, process-related impurities, and soluble spike proteins variants could result in conformational changes of PF-4 [29]. The proinflammatory milieu caused by vaccine components consisting of human cell line proteins, free virus proteins, EDTA, and AdV genetic material may facilitate the immune response of anti-PF4. Another point is that soluble spike protein variants produced by alternative splicing post ChAdOx1 vaccine can induce damage of endothelial cells and subsequent thrombosis by binding to ACE2 [29]. Additionally, when compared to mRNA vaccination, thrombin generation was higher followingChAdOx1 vaccination, suggesting that specific components of the vaccination may contribute to the development of VITT [30]. Sverre Holm et al. investigated the pathological immune responses in patients with VITT [31], and the result showed that the circulating immune complex could activate platelets directly and contained multiple innate immune pathway triggers. Moreover, inflammatory markers such as interleukin-6 (IL-6), interleukin-18 (IL-18), soluble CD163 (sCD163), lipopolysaccharide-binding protein (LBP), P-selectin levels, and neutrophil extracellular traposis (NETosis) markers were significantly elevated. Anti-PF4/polyanion IgG and PF4/polyanion form immune complexes, which activate platelets via IgG/FccRIIA. Of note, the thrombus formation is accompanied by a significant activation of the innate immune system, especially the activation of neutrophils and NETosis. Another postulated cofactor of VITT is procoagulant microparticles (MPs), which are derived from platelets and monocytes expressing phosphatidylserine and tissue factor [32]. The circulating MPs promote the formation of thrombus. 

#### 3.2.3. Management of VITT

Individuals presenting with persistent headache, abdominal pain, limb pain/swelling, and neurologic symptoms, etc. following vaccination within 5 to 30 days should be suspected as having VITT [33]. Laboratory tests include platelet counts, D-dimer, fibrinogen, international normalized ratio/prothrombin time, activated partial thromboplastin time, and PF4 antibodies. In patients suspected of splanchnic vein thrombosis, bilirubin, aspartate transaminase, alanine transaminase, and alkaline phosphatase can be helpful. Of note, platelet counts and D-dimer comprise the most important laboratory tests for screening [34]. Appropriate imaging should be selected according to the suspected site of the thrombosis. For example, when patients present with limb pain or swelling, a color Doppler ultrasonography is recommended. For patients suspected of CVST, CT venography or MRV is an appropriate choice. In addition, an intravenous, contrast-enhanced CT scan is an option for patients with suspected splanchnic vein thrombosis.

Therapeutic anticoagulation and high-dose intravenous immunoglobin (IVIG) are recognized as first-line therapies for VITT. In patients with aHIT, the administration of IVIG can rapidly increase platelet count and reduce hypercoagulability. Since platelet activation may be a critical pathogenesis of VITT, and several case series reported that IVIG improved platelet count in VITT patients; several professional societies recommend the early use of IVIG in patients with a confirmed diagnosis or high suspicion of VITT [35]. Non-heparin anticoagulants such as direct oral anticoagulants (dabigatran, apixaban, rivaroxaban, edoxaban) and direct thrombin inhibitors (bivalirudin and argatroban) are recommended. The duration of the anticoagulation interval is unknown, but at least three months is recommended [35]. Vitamin K antagonists should be avoided in acute VITT according to the clinical experience of the management of HIT. Patients with HIT treated with vitamin K antagonists have an increased risk for the development of venous limb gangrene and skin necrosis. The actual risk of warfarin-associated venous limb gangrene is approximately 5–20% [36]. However, there is controversy over the use of heparin treatment. In a meta-analysis [24], when compared to the non-heparin anticoagulants group, no significant difference was found in heparin-based therapy. Avoidance of heparin may be due to the difficulty in ruling out heparin-related cross-reactions. IVIG can inhibit platelet activation and aggregation. The recommended dose for IVIG is 1g/kg initially and 1g/kg again on day 2 for patients with splanchnic thrombosis and CVST. If IVIG is not available, corticosteroids may be used as an alternative [34]. Moreover, plasma exchange can quickly remove pathological antibodies from circulation. Other therapies include rituximab (to decrease new antibody production targeting CD20-positive B cells) and eculizumab (to inhibit thromboembolic responses targeting complement C5). Transfusions with platelet and fibrinogen are not routine treatments and should be used with caution and only considered in selected patients such as patients with severe bleeding, severe thrombocytopenia, and surgical intervention [36]. The management of VITT is summarized in Table 2.

#### 3.2.4. Immune Thrombocytopenia

Immune thrombocytopenia (ITP) is an autoimmune disorder with the destruction and impaired production of platelets, resulting in a low platelet count. Secondary ITP is defined as autoimmune thrombocytopenia that occurs in the context of other disease conditions such as infections, other autoimmune diseases, and hematologic malignancies [37]. Cases of ITP associated with several vaccines have been reported, including the influenza vaccine, poliomyelitis vaccine, etc. Although thrombocytopenia has not been recognized as a common side effect in clinical trials, cases of ITP or acute exacerbation of preexisting chronic ITP after COVID-19 vaccination have been reported.

C. R. Simpson et al. observed an association between ChAdOx1 vaccination and ITP, with an adjusted rate ratio of 5.77 (95% CI, 2.41–13.83) within 0 to 27 days after vaccination, suggesting an incidence of 1.13 (0.62–1.63) cases per 100,000 doses. They found no association between the BNT162b2 vaccine and ITP [38]. However, using the data from the Vaccine Adverse Events Reporting System (VAERS), 77 patients suspected with de novo ITP after COVID-19 vaccination (BNT162b2 or mRNA-1273) were identified, with a platelet count of 3 (0–9) × 10^9/L [39]. In one study, including 66 patients with ITP post anti-SARS-CoV-2 vaccines [40], 16 patients had preexisting ITP. Regarding demographic characteristics, the mean age was 63 years, and females accounted for 60.6% of cases. More ITP events happened after mRNA vaccines (BNT162b2 or mRNA-1273) compared to after adenoviral vaccines (ChAdOx1-S or Ad26.COV2-S). Moreover, the average time from vaccination to onset of symptoms was 8.4 days. Of the patients, 73% and 27% developed symptoms after the first dose and the second dose of vaccination, respectively.

The possible mechanism of mRNA-based (BNT162b2 or mRNA-1273) vaccine-related ITP remains unclear, but may be due to the mRNA. mRNA uptake by cellular receptors leads to the activation of immune cells. Activated immune cells secrete cytokines and chemokines, contributing to autoimmune disorders. In addition, lipid nanoparticles can foster immune activation, which is a component of mRNA vaccines [41]. After the administration of anti-SARS-CoV-2 vaccines, protective antibodies are produced, and these antibodies possibly target antigens on the surface of platelets, which is called molecular mimicry [40]. Moreover, non-mRNA-based COVID-19 vaccines can trigger ITP. The main compounds of the vaccines such as the viral vector, attenuated virus, and viral protein may activate autoimmune pathways. In addition, the adjuvants and preservatives of vaccines may induce autoimmune responses, thus, contributing to the development of ITP [41]. 

The most common therapies for ITP following COVID-19 vaccination are steroids and IVIG. Other drugs used include rituximab, thrombopoietin receptor agonists, and vincristine [39]. Estimated efficacy of treatment is 90%, and the outcome is usually positive [42]. Treatments for ITP and exacerbation of preexisting ITP following COVID-19 vaccination are similar to those which are used in cases that do not follow vaccination.

#### 3.2.5. Thrombotic Thrombocytopenic Purpura

Thrombotic thrombocytopenic purpura (TTP) is a rare thrombotic microangiopathy. Immune-mediated TTP (iTTP) is characterized by autoimmune antibodies against ADAMTS13, a cleaving protease for von Willebrand factor. Severe deficiency of ADAMTS13 leads to microangiopathic hemolytic anemia, consumptive thrombocytopenia, and organ damage. Cases of iTTP following COVID-19 vaccination have been reported. Laboratory tests revealed low ADAMTS13 levels and high titer of anti-ADAMTS13 antibodies [43]. 

A recent study included 27 cases of TTP (de novo or relapse) after COVID-19 vaccines, and the result showed that TTP episodes were more frequently observed post BNT162b2 vaccine, followed by post mRNA-1273 vaccine. Mucocutaneous bleeding was the most common clinical manifestation. The mean days from vaccine immunization to the onset of symptoms were 13.4 days [44]. Therapies included plasma exchange, caplacizumab, rituximab, and corticosteroids. For iTTP that occurs after vaccination, the current TTP guidelines are recommended for management. iTTP should be distinguished from VITT, though they share some similar characteristics, such as neurological presentation and thrombocytopenia [45]. Levels of D-dimers, fibrinogen, and laboratory tests for microangiopathic hemolytic anemia can help distinguish TTP from VITT. In addition, ADAMTS13 activity, anti-ADAMTS13 antibodies, and anti-PF4 antibodies can facilitate diagnosis.

To date, it is unclear whether there is a clear causal relationship between the COVID-19 vaccine and iTTP. However, vaccination may trigger iTTP in patients with a preexisting deficiency of ADAMTS13. It is known that severe ADAMTS13 deficiency is not enough to cause clinically detectable symptoms unless triggered by other risk factors. Therefore, it is speculated that the vaccine may cause endothelial damage, which contributes to the onset of TTP [45]. Further studies are required to reveal the potential mechanisms of iTTP and COVID-19 vaccines.

### 3.3. Hemorrhage

Hemorrhagic events are often related to thrombosis and thrombocytopenia. Intracerebral hemorrhage and or subarachnoid hemorrhage are observed in 49% of patients with CVST and VITT [25]. Patients with CVT can suffer bleeding in the brain parenchyma. The possible mechanism is that, once the vessel is blocked, the increased pressure can lead to the rupture of the fragile vessel and subsequent bleeding [13]. Other sites of bleeding events include vaginal bleeding, subungual hematomas, etc.

Acquired hemophilia A is a life-threatening bleeding disorder. The autoantibodies against coagulation factor VIII (FVIII) lead to decreased activity and/or accelerated clearance of FVIII. AHA cases related to SARS-CoV-2 vaccines have been reported. Massimo Franchini et al. conducted a disproportionality analysis and systematic case review using the data from VigiBase, VAERS, and a literature search. The result showed that the information component (IC) was significant for the association of AHA with all SARS-CoV-2 vaccines and BNT162b2 with an IC025 at 1.1 and 1.6, respectively, suggesting that the signal of AHA associated with COVID-19 vaccination is robust [46]. Approximately 22% of cases occurred in individuals younger than 65 years, with a mortality of 11%. Moreover, there were no pregnancy-related cases. The median time to diagnosis was 18 days. Of the cases, 40% developed after the second dose. Of note, no predispositions to AHA were detected in approximately 57% of the cases [46]. The underlying mechanism of AHA after COVID-19 vaccination remains unknown. FVIII inhibition using anti-SARS-CoV2-spike-IgG (anti-S-IgG) was weak, which suggests that cross-reactivity induced by vaccination is likely not the trigger of FVIII inhibition. Additionally, mRNA vaccines are known to be associated with the upregulation of toll-like receptor, which may stimulate immune cell activation, contributing to autoantibody production in individuals predisposed to AHA [47]. Treatments include steroids, recombinant activated clotting factor VII, immunosuppressive therapy (cyclophosphamide), and rituximab [48]. Extremely rare cases of another coagulation factor inhibition, acquired factor XIII (F13) deficiency, have also been reported following COVID-19 vaccination [49]. Therapies include F13 concentrates, prednisolone, and IVIG.

### 3.4. Other Hematological Events

Hemolytic anemia after COVID-19 vaccination has been observed. Autoimmune hemolytic anemia (AIHA) is characterized by autoantibodies against erythrocytes causing the destruction of red blood cells. AIHA can be divided into warm type and cold type. In a study [50], 18 cases of AIHA (11 newly developed AIHA cases and seven exacerbated AIHA cases) following COVID-19 vaccination were included. Among the new-onset AIHA cases, warm IgG was detected in 9 of 11 cases, and cold IgM was observed in one case. In addition, mixed autoantibodies were described in one case. Most of the cases received mRNA vaccines. The median age of patients was 67 years. The median days to onset of symptoms following vaccination were 7 and 14 days for the first dose and second dose, respectively. Among the exacerbated AIHA cases, three developed IgG, and four developed IgM autoantibodies. The median age was 73 years. Treatments for the reported patients included blood transfusions, steroids, rituximab, recombinant erythropoietin, and mycophenolate mofetil [51].

Other rare hematological events associated with COVID-19 vaccination, such as paroxysmal nocturnal hemoglobinuria (PNH) [52], hemophagocytic lymphohistiocytosis [53], and Evans syndrome, have also been reported [54]. These conditions are extremely rare and have only been seen in a small number of case reports. PNH is a complement-mediated hematologic disorder. Cases of pharmacodynamic breakthrough in PNH patients treated with ravulizumab (a complement inhibitor for C5) have been observed after COVID-19 vaccination. Stimulation of the lectin pathway by the SARS-CoV-2 spike protein and subsequent unrestrained complement activation have been recognized as a possible mechanism causing inflammation, endothelial cell dysfunction, multiorgan failure and even death in COVID-19 [55]. Unlike this, PNH exacerbation appears to occur independently of the spike protein and may be associated with inflammation triggered by adjuvants in the vaccine, since this protein cannot induce PNH erythrocytes lysis in vitro [56]. Evans syndrome is defined as the simultaneous existence of AIHA and ITP. Secondary Evans syndrome can be triggered by autoimmune diseases in particular systemic lupus erythematosus, infections, and hematologic malignancies. In this case, the patient develops new-onset Evans syndrome associated with SLE following mRNA vaccination [54]. Little is known about the underlying mechanisms.

## 4. COVID-19 Vaccination in Patients with Hematological Disorders

### 4.1. Vaccination in Patients with VITT

In individuals confirmed with VITT, it is recommended that full vaccination should be given if mRNA vaccines are available. Regarding the type of second-dose vaccination, mRNA vaccines are safe choices. It is likely that inactivated virus vaccines are also safe, although there is no available data. Moreover, most VITT patients would tolerate a second dose of ChAdOx1 nCov-19 vaccine, and VITT is recognized as a problem almost exclusively associated with the first dose by Andreas Greinacher et al. according to the fact that most cases reported after a second dose had negative PF4 antibodies [34].

### 4.2. Vaccination in Patients with ITP

Since ITP has been described following COVID-19 vaccination, there is concern about whether COVID-19 vaccination can exacerbate preexisting ITP. However, little data are available regarding the incidence of ITP exacerbation after COVID-19 vaccination in individuals with previously existing chronic or persistent ITP. A total of 34 patients with chronic or persistent ITP were included in a retrospective observational study, and platelet counts were recorded before and following COVID-19 vaccination (58.8% received BNT162b2, and 41.2% received mRNA-1273). The results showed that platelet counts decreased by 20% in 47.1% of patients following the first dose of vaccination. In addition, after dose 2, 44.1% had decreased platelet counts. No significant difference was observed between the two vaccines. Overall, platelet decreases were transient and responded well to treatment [57]. A total of 52 consecutive patients with chronic ITP were included for a prospective investigation after COVID-19 vaccination [58]. Of the patients, 12% were observed to have new bleeding symptoms with a median platelet count drop of 96% 2 to 5 days after vaccination. Of the patients, 73% presented with no deteriorated ITP symptoms and no significantly decreased platelet counts. The result suggests that patients with previously existing ITP may suffer an exacerbation of thrombocytopenia; however, the outcome for this thrombocytopenia seems positive after treatment with corticosteroids and or IVIG. In addition, the occurrence of ITP exacerbation was independent of remission status, concurrent ITP treatment, or vaccine type [58]. In another study [59], 218 ITP patients and 200 healthy controls were included. A 6.3% decrease in platelet counts was found in ITP and healthy groups. Nevertheless, no significant difference was observed between the two groups. Baseline platelet count < 50 × 10^9^/L (OR, 5.3; 95% CI, 2.1–13.7), under treatment for ITP (OR, 3.4; 95% CI, 1.5–8.0) and age (OR, 0.96 per year; 95% CI, 0.94–0.99) were risk factors for the development of ITP exacerbation.

It was recommended by David J. Kuter et al. that patients with ITP receive at least the first dose of the COVID-19 vaccination [58]. Of note, clinicians should keep in mind that worsening thrombocytopenia might develop in this group following vaccination, and close monitoring of platelet counts may be required in these patients. Further studies are needed to clarify the exact association between COVID-19 vaccination and ITP.

### 4.3. Vaccination in Patients with TTP

Immune-mediated thrombotic thrombocytopenic purpura following SARS-CoV-2 vaccination has been reported, which has led to safety concerns about COVID-19 vaccination in patients with a history of iTTP. Whether vaccination is associated with a recurrence of the disease is unknown. Among 79 patients with iTTP immunized with COVID-19 vaccines, one case (1.3%) suffered iTTP relapse. The patient received the second dose of the vaccine when her ADAMTS13 activity was >20% and showed a promising outcome. The estimated incidence of iTTP relapse following immunization was 0.095 per patient-years in this study [60]. Another prospective cohort study included 32 preexisting iTTP patients who received mRNA COVID-19 vaccination (30 received Pfizer/BioNTech, and two received Moderna). A total of five patients had no detectable ADAMTS13 activity after vaccination with a median time of 15 days following vaccination [61]. 

Although iTTP relapse may rarely occur, Gaetano Giuffrida et al. concluded that the benefits of vaccination may outweigh the risk of recurrence. Of note, in this condition, close monitoring of blood routine, ADAMTS13 activity, and anti-ADAMTS13 antibodies following COVID-19 vaccination can contribute to early diagnosis of relapses [61]. Additional research is needed to reveal the association between iTTP and COVID-19 vaccines.

### 4.4. Vaccination in Patients with Hematologic Malignancies

Patients with malignancy are susceptible to SARS-CoV-2 infection and have high mortality and morbidity following infection. COVID-19 vaccination is recommended for patients with tumors, particularly those with hematologic malignancies [62]. In a retrospective analysis [63], Evgenii Shumilov et al. aimed to compare the clinical course of COVID-19 in cancer patients between vaccinated and non-vaccinated groups. In the vaccinated group, a mild course of COVID-19 was more common (49% vs. 29%), and patients with hematologic tumors in particular benefited from vaccination. A total of 1548 patients with hematological malignancy who were diagnosed with breakthrough COVID-19 after receiving at least one dose of COVID-19 vaccination were included in this analysis, and the mortality rate was significantly lower than that reported in the pre-vaccine era (pre-vaccine 31.2% vs. post-vaccine 9.2%; *p* < 0.001) [64].

However, a lower antibody response against COVID-19 immunization compared to that of healthy individuals may limit the efficacy of vaccines in patients with hematologic malignancies. In a meta-analysis including 7064 patients with hematologic malignancies following COVID-19 vaccination [65], overall, seropositivity rates after one and two doses of the COVID-19 vaccine were 37–51% and 62–66%, respectively. Moreover, patients with chronic lymphocytic leukemia had the lowest seropositivity rate, while patients with acute leukemia had the highest seropositivity rate. Response rates of neutralizing antibodies and cells were 57–60% and 40–75%, respectively. CD20 monoclonal antibody therapies, more profound immunosuppression, and age were associated with poor immune responses against COVID-19 vaccination. Of note, higher antibody levels were observed in patients immunized with mRNA-1273 compared to BTN162b2 or adenoviral-based vaccines, even adjusted for other factors [66]. The serum antibodies against spike subunit 1 were detected in healthy controls following the standard two-dose vaccination with mRNA-1273 and in patients with hematologic cancers following one additional mRNA-1273 vaccination after the standard two doses. The result revealed comparable median antibody concentrations between the two groups, suggesting that a supplementary third vaccination for patients with immunosuppression and hematologic cancers could be favorable [67].

In general, risk factors associated with reduced antibody response following COVID-19 vaccination in patients with hematological malignancies include patient-specific and vaccine-specific factors. Patients with lymphoproliferative disorders, active disease, older age, immunosuppression, and male sex have reduced antibody responses against SARS-CoV-2 vaccination. The inhibitory effect was most obviously seen in B-cell-depleting therapies such as CD20-targeted therapies. Regarding vaccine-specific factors, mRNA vaccines seem to be a better choice, with a higher antibody response compared to adenovirus-based vaccines, particularly the mRNA-1273 vaccine [68]. In addition, it is likely that booster vaccination can increase immune responses among these patients. Of note, the safety of COVID-19 vaccination is similar to that of the healthy population. Due to the impaired responses against COVID-19 vaccination in patients with hematologic malignancies, additional protective strategies are required. Vaccinating close contacts is an effective means of protecting susceptible individuals [68]. 

## 5. Conclusions

Vaccination plays a crucial role in controlling the spread of SARS-CoV-2, thus, quelling the pandemic of COVID-19. Despite cases of rare adverse events associated with COVID-19 vaccination having been reported and raising concerns, the overall benefits of immunization outweigh the risks. Vaccination is a safe and effective tool against severe COVID-19. However, clinicians should be aware of these adverse events. Once an event happens, prompt diagnosis and management are essential. In addition, the mechanisms remain unclear and require further investigation. Regarding vaccination in individuals with preexisting hematological disorders, the effect of COVID-19 vaccination on these patients has not been well characterized. More investigations are required to develop a better vaccination schedule and appropriate post-vaccine surveillance plans.

## Figures and Tables

**Table 1:** The features of COVID-19 vaccines commonly used.

Platforms	Mechanism	Vaccine	Trade Name	Manufacturer	Components
Inactivated	the entire virus as an immunogen triggers immune responses and leads to the production of a wide spectrum of antibodies	BBIBP-CorV	Covilo	Sinopharm (Beijing)	β-propanolide-inactivated virus with alum adjuvant;
		PiCoVacc	CoronaVac	Sinovac Biotech	Vero cell-propagated, β-propiolactone-inactivated virus with alum adjuvant;
		BBV152	Covaxin	Bharat Biotech	Whole inactivated virus with Algel-IMDG adjuvant.
mRNA	trigger Th1 cell responses, trigger germinal center B cell responses	BNT162b2	Comirnaty	Pfizer/BioNTech	Lipid nanoparticle-encapsulated mRNA encoding S-2P antigen;
		mRNA-1273	Spikevax	Moderna	Lipid nanoparticle-encapsulated mRNA encoding S-2P antigen.
Viral vector	trigger Th1 cell responses	ChAdOx1 nCoV-19, AZD1222	Vaxzevria	AstraZeneca/Oxford	Recombinant chimpanzee adenovirus vector (ChAdOx1) encoding the spike protein antigen of SARS-CoV-2;
		Ad26.COV2.S	-	Johnson & Johnson	Non-replicating human adenovirus vector (Ad26) expressing S-2P antigen;
		Ad5-nCoV	Convidecia	CanSino Biologics	Non-replicating human adenovirus vector (Ad5) expressing wildtype S protein;
		Gam-COVID-Vac	Sputnik V	Gamaleya Research Institute	Non-replicating adenovirus (combination of Ad26 and Ad5) expressing wildtype S protein.
Protein subunit	trigger Th1 cell responses	NVX-CoV2373	Nuvaxovid	Novavax	Recombinant nanoparticle prefusion spike protein formulated with Matrix-M adjuvant.

Modified from references [5,6]. S-2P: full-length SARS-CoV-2 spike protein with two proline substitutions at residues K986 and V987, S protein: spike protein of SARS-CoV-2.

**Table 2:** Management of VITT.

1.Vaccination history
Onset of symptoms 5–30 days following vaccination (in particular, received adenovirus vector-based vaccines).
2. Warning symptoms
Severe, persistent, or recurrent headaches, abdominal pain, shortness of breath, chest pain, limb pain/swelling, etc.
3. Laboratory tests
Platelet count, D-dimer, INR/PT, APTT, fibrinogen;
Antibody testing: platelet factor 4 antibody tests;
If splanchnic vein thrombosis is suspected: Bilirubin, aspartate transaminase, alanine transaminase, alkaline phosphatase.
4. Imaging examination
Select according to the suspected site of the thrombosis.
5.Traetment
High-dose IVIG: 1g/kg initially, 1g/kg again on day 2 for patients with splanchnic thrombosis and CVST;
Non-heparin anticoagulants: DOACs (dabigatran, apixaban, rivaroxaban, edoxaban), direct thrombin inhibitors (bivalirudin and argatroban), fondaparinux;
If IVIG is not available, corticosteroids may be used as an alternative;
Avoid vitamin K antagonists and aspirin;
Plasma exchange;
Investigational therapies: rituximab, eculizumab.

VITT: vaccine-induced immune thrombotic thrombocytopenia, CVST: cerebral venous sinus thrombosis, DVT: deep vein thrombosis, PE: pulmonary embolism, INR: international normalized ratio, PT: prothrombin time, APTT: activated partial thromboplastin time, IVIG: intravenous immunoglobulin, DOACs: direct oral anticoagulants.

## Data Availability

Not applicable.
